# The Utility of Lower Glycemic Targets for Treating Gestational Diabetes: A Retrospective Study

**DOI:** 10.1155/2019/6372474

**Published:** 2019-12-05

**Authors:** Grace Hagen, Crystal Brown, Jordan Dietrich, Charles Gibbs, Gene T. Lee

**Affiliations:** Department of OBGYN, University of Kansas Medical Center, Kansas City, KS, USA

## Abstract

**Objective:**

In vivo study of glucose homeostasis in pregnancy suggests normal glucose levels are lower than current glycemic targets used in gestational diabetes. After the HAPO study results, our institution began using glycemic targets of fasting 85 mg/dL and 2-hour postprandial of 110 mg/dL. We reviewed our results.

**Methods:**

A retrospective cohort of GDM patients that delivered at KUMC from January 2007 to May 2017 was reviewed. All patients were diagnosed with the 2-step Carpenter-Coustan thresholds. High targets were compared with low targets. The primary outcome investigated was birthweight > 90% (large for gestational age, LGA).

**Results:**

604 patients were studied, and 34% were treated with low glycemic targets. Our unadjusted results showed that the low-target group had a lower incidence of LGA infants (24.0 vs. 31.8%), higher incidence of neonatal hypoglycemia (20.7 vs. 11.6%), and inductions (39.4 vs. 20.5%). After adjustment for demographic variables, only a higher risk of inductions remained (aOR 2.54 (1.44, 4.49)).

**Conclusion:**

Lower glycemic targets did not produce large reductions in fetal overgrowth, but they were associated with a higher rate of inductions. As there were no observed differences in maternal or neonatal outcomes otherwise, aiming for lower glycemic targets in GDM is likely not cost-effective.

## 1. Introduction

Gestational diabetes (GDM) is a frequent condition in otherwise normal pregnancies which leads to major morbidity for both the mother and the baby [1]. Complications can be mild, such as macrosomia or polyhydramnios, or severe, such as cesarean deliveries or fetal shoulder dystocia. For optimal outcomes, it is clear that GDM should be identified, monitored, and treated in pregnancy [2, 3]. However, the glycemic targets that patients and clinicians should use are not supported by solid evidence [4, 5]. Leaving this question unanswered means that patients will continue to experience morbidity unnecessarily.

The current targets recommended by most organizations are a fasting < 95 mg/dL and a 2-hour postprandial < 120 mg/dL [1, 6]. These targets may be too high if the goal is to approximate normal levels in pregnancy. Both the Hyperglycemia and Adverse Pregnancy Outcomes Study (HAPO) and a review of normal glycemic levels in uncomplicated pregnancies suggest that the mean is much lower than the current thresholds [4, 7].

Since early 2011, our department has aimed for lower targets based on our understanding of normal physiology in pregnancy and the HAPO study. Because there was no threshold effect seen in the HAPO results, we simply chose to lower the recommended glycemic targets by 10 mg/dL. Thus, our targets became a fasting of less than 85 mg/dL and a 2-hour postprandial of less than 110 mg/dL. The objective of this study is to evaluate whether our adoption of lower glycemic targets led to better outcomes for patients with GDM. We hypothesized that the lower glycemic targets would result in lower morbidity in our GDM patients.

## 2. Materials and Methods

We conducted a retrospective chart review of pregnant women diagnosed with GDM at our medical center between January 2007 and May 2017. This study was included under IRB protocol #3506, initially approved 12/29/2015. Patients were identified by manual review of delivery logs, electronic query of our ultrasound database for a diagnosis of GDM, and electronic query of our electronic medical record for a diagnosis of GDM. Deliveries between February 16, 2011, and May 4, 2011, were excluded as there was a transition period where patients were treated with 2 sets of glycemic targets. Inclusion criteria were age > 16 years, singleton pregnancy, and diagnosis of GDM using the 2-step approach. Exclusion criteria were multiple gestations, preexisting diabetes, congenital anomalies, and significant maternal chronic disease such as cardiac disease, renal insufficiency or nephrotic condition, systemic lupus erythematosus, severe asthma, uncontrolled thyroid disease, chronic hepatitis, HIV infection, or neurologic condition. Chronic hypertension was specifically not excluded as we felt that it was a common comorbidity present in patients with obesity and impaired glucose tolerance who are at high risk for GDM.

All patients were diagnosed with the 2-step approach using Carpenter-Coustan thresholds. This approach included an initial screen with a 50-gram oral glucose load between 24 and 28 weeks of gestation. After 1 hour, if the serum glucose reported ≥140 mg/dL, a diagnostic 3-hour 100-gram glucose tolerance test was administered. Patients were defined as GDM if 2 or more values were abnormal (fasting ≥ 95 mg/dL, 1‐hour ≥ 180 mg/dL, 2‐hour ≥ 155 mg/dL, and 3‐hour ≥ 140 mg/dL). Patients with risk factors for diabetes (obesity, family history, ethnicity, and prior GDM) were also screened during the first trimester or at their first prenatal visit with the 50-gram glucose load. Diagnostic testing occurred whenever indicated. Patients whose fasting glucose ≥ 126 mg/dL or HgbA1c ≥ 6.5 in the first trimester were considered to have preexisting diabetes, and these patients were excluded.

Prior to February 16, 2011, glycemic targets for treatment of GDM in our high-risk pregnancy clinic were consistent with guidelines from ACOG and American Diabetes Association [1, 6]. They were fasting glucose < 95 mg/dL and 2-hour postprandial < 120 mg/dL. Patients prior to this date were verified by personal review of medical chart using these conventional glycemic targets. This historical control group was labeled as “high” glycemic targets. The new lower targets of fasting < 85 mg/dL and 2-hour postprandial < 110 mg/dL were introduced on a single day and applied to all new and existing GDM patients. Allowing for a washout period of 11 weeks, patients who delivered after May 4, 2011, had only used the new lower targets and were grouped as using “low” glycemic targets. Again, personal review of medical charts verified the lower glycemic targets used in their treatment of GDM.

Management of GDM patients was uniform in the department. All GDM patients were referred to the high-risk pregnancy clinic staffed by perinatologists. A small number of patients were allowed to be comanaged with their original OBGYN if their glucose control was excellent and their treatment only uses oral medications. When more than fifty percent of glucose values at any given time point were elevated, oral medical treatment with metformin or glyburide is started. Doses were increased weekly if glucose levels were not controlled. If glucose levels were not controlled despite maximal doses of oral medications, subcutaneous insulin was started. Hemoglobin A1c values were not regularly used to guide treatment. A nurse and dietitian both provided diabetes education. Patients are instructed to eat regular portions as consistently as possible, with carbohydrate goals of 30-45 grams for breakfast, 45-60 grams for lunch, and 45-60 grams for dinner. GDM patients on medications were induced at 39w0d if their labor had not started spontaneously. GDM patients controlled by diet were expectantly managed until 40-41 weeks. Intrapartum protocols for diabetes patients include glucose monitoring every 2-4 hours during the latent phase and every 1-2 hours in the active phase and using insulin subcutaneously or intravenously to keep glucose < 120 mg/dL.

The primary outcome investigated was birthweight > 90% (large for gestational age, LGA) [8–10]. Secondary neonatal outcomes included macrosomia (birthweight > 4000 grams), birthweight, hypoglycemia (glucose < 40 mg/dL), phototherapy for hyperbilirubinemia, NICU admission, respiratory distress, and death. Secondary obstetrical outcomes included cesarean delivery, preterm delivery, preeclampsia, shoulder dystocia, and stillbirth. Because chronic hypertension is a known risk factor for low birthweight infants, a subgroup analysis was planned where patients with chronic hypertension were excluded.

SPSS statistical software was used to analyze data, including chi-square tests, *t*-tests, and *Z*-tests, as needed. Results were analyzed by logistic and ordinary least squares regression due to observed demographic differences between the groups. A bivariate analysis was used to identify significant variables for inclusion in the regression models. Adjusted odds ratios (aOR) as well as 95% confidence intervals (95% CI) are reported. A *p* value below 0.05 and odds ratios which do not cross 1 were considered statistically significant.

Study size was determined using the incidence of LGA in GDM pregnancies from prior studies from our institution [11]. With a 30-35% incidence of LGA, we calculated that 600 patients with 2 equal groups would have sufficient power to detect a 33% reduction in the primary outcome.

## 3. Results

We reviewed 793 pregnancies with GDM, and 604 charts were available for analysis. These 604 charts had complete information regarding delivery outcome, birthweight, and neonatal course. 189 charts were excluded, and the largest reason for exclusion was nonretrievable or missing medical chart (85/793, 10.7%). The other categories included the following: congenital anomalies (*n* = 29), multiple gestation (*n* = 14), significant maternal chronic disease (*n* = 22), GDM diagnosed by the one-step approach (*n* = 10), and GDM pregnancies which occurred during the transition period (*n* = 29).

There were demographic differences between the groups (Table 1). The low-target group showed a lower proportion of Hispanic ethnicity (33 vs. 59%) and lower rates of prior GDM history (14 vs. 23%). The initial Glucola screen was higher (175 vs. 167 mg/dL). During the diagnostic glucose tolerance test, the fasting value was lower (93.7 vs. 97.6 mg/dL), but the subsequent values were similar.

Measured outcomes initially showed a lower rate of LGA infants (24 vs. 31.8%) along with a higher rate of neonatal hypoglycemia (20.7 vs. 11.6%) and inductions (39.4 vs. 20.5%) in the low-target group (Table 2).

A bivariate analysis identified nulliparity, BMI at intake, Hispanic ethnicity, and prior GDM history as important confounding variables. After adjustment for these characteristics, a significant effect of the low-target group was found for the outcome of inductions, aOR 2.54 (1.44, 4.49). The other outcomes of LGA, macrosomia, and neonatal hypoglycemia did not show a significant effect with the low-target group (Table 3). Our subanalysis excluding patients with chronic hypertension did not change our prior findings.

## 4. Discussion

Our study did not find that lower glycemic targets result in improved outcomes for pregnancies complicated by GDM. Our aggregate findings showed a possible reduction in LGA incidence, but demographic characteristics such as Hispanic ethnicity, prior GDM diagnosis, BMI, and nulliparity removed the statistical significance once multivariable regression was performed. In contrast, our study suggests that a higher number of inductions are seen when patients are asked to pursue low targets for their GDM care.

Our practice adopted lower glycemic targets based on the assumption that a glucose profile as close to euglycemia as possible would have the best outcomes. Several indirect lines of evidence support our assumption. First, results from the HAPO study showed that outcomes of macrosomia, cesarean delivery, and increased cord insulin levels increased with each stepwise increase in glucose recorded after the administration of a 75-gram 2-hour glucose tolerance test [7]. However, this study only established the association between the results of a one-time diagnostic test and health outcomes. A clearer association between glycemia and morbidity would have to report average glycemic levels over a duration of time. Second, data from inpatient and continuous glucose monitors show that the average fasting and postprandial glucose levels in pregnant women without obesity nor impaired glucose tolerance were much lower than previously believed [4]. The reported average values were fasting of 71 ± 8 mg/dL, 1-hour postprandial as 109 ± 13 mg/dL, 2-hour postprandial as 99 ± 10 mg/dL, and 24 h glucose as 88 ± 10 mg/dL. Third, modern continuous glucose monitoring technology has demonstrated that GDM pregnancies have occult periods of hyperglycemia not captured using fingerstick monitoring [12]. All of these findings suggest that unaccounted or occult glycemia could account for persisting morbidity seen in well-controlled GDM patients [13].

One explanation for our null findings with regard to LGA and macrosomia is that the lower glycemic targets were applied universally rather than selectively to our GDM patients. A 2016 Cochrane study attempted to study whether lower glycemic targets would improve outcomes in GDM pregnancies [10]. Only one abstract presented at a conference was found, and no differences were found. In contrast, a series of randomized trials exists where lower glycemic targets were selectively applied only to those GDM pregnancies where a fetal abdominal circumference in the third trimester suggested overgrowth [14–18]. In these studies, patients whose fetus showed an abdominal circumference < 75% were allowed to have relaxed glycemic targets such as fasting 100 mg/dL and 2-hour postprandial 140 mg/dL [18] or even 120/200 [17]. In contrast, fetuses with evidence of accelerated fetal growth were instructed to aim for lower glycemic thresholds, 80/100 or 80/110. In these clinical trials, investigators found that lower rates of LGA infants could be achieved with lower overall rates of insulin treatment. Lower targets may only be effective when directed at particularly high-risk subsets within GDM patients. A study consistent with this concept would include the randomized trial of mild GDM pregnancies by Casey et al. [19]. In this trial, mild GDM patients were defined as having a fasting glucose < 105 mg/dL. Subjects were randomized to either diet or diet plus glyburide. Although the glycemic profiles improved in the group treated with glyburide, the maternal and neonatal outcomes did not differ.

Another explanation for the absence of differences in LGA or macrosomia is that patients did not actually achieve their lower glycemic targets. Unfortunately, this study did not have complete data regarding compliance or medications as glucose logs from the earlier years in the cohort were not available. In addition, our practice does not regularly check hemoglobin A1c levels either. Nevertheless, this study reflects a natural history experiment in real-world clinical practice. One major obstacle in aiming for low-glycemic targets may be that patients or physicians may be unable or unwilling to achieve them. Asking patients to achieve lower glycemic targets may not be feasible outside of tightly controlled study conditions. Perhaps future studies may be able to capitalize on technologies such as continuous glucose monitors to demonstrate achievement of lower glycemic averages and subsequent improved outcomes.

The finding of a higher rate of inductions was a secondary outcome of this study, but it suggests that aiming for lower glycemic targets may not be cost-effective. After all, if no differences are found in maternal or neonatal outcomes, then the extra costs associated with a greater number of inductions are not justified [20]. We hypothesize that the greater number of inductions occurred because the low-target group had a greater proportion of patients on oral medication. A previously published study at our institution found that adoption of the one-step approach for diagnosing GDM not only increased the number of GDM patients but also increased the number of GDM patients treated with oral medication [11]. Regrettably, our dataset for this study extended further into the past and did not record medication prescribed to each GDM patient.

Another hypothesis for the increased rate of inductions is that hyperglycemia leads to earlier onset of labor [21]. For example, higher hemoglobin A1c values are associated with higher risk for preterm delivery in persons with type 1 diabetes [22]. Pregnancies with poorly controlled GDM have also been shown to have higher rates of preterm delivery compared to normal pregnancies [23]. Lastly, higher average glucose values are seen in normal pregnancies who experience spontaneous preterm delivery compared to controls with spontaneous term labor [24]. Applied to our findings, the above hypothesis would argue that the lower glycemic target group allowed more GDM pregnancies to reach 39 weeks, at which point they were recommended for induction of labor.

Our study is limited in several ways. First, it is a retrospective study and has the issue of confounding. At the same time that our department adopted the lower glycemic targets, we also allowed faculty to self-select either the one-step or two-step approach for GDM screening [11]. We only analyzed those patients who were screened with the two-step approach, but the observed differences in Hispanic ethnicity and prior GDM rates likely are a consequence of providers' bias for which patients were screened with the 2-step approach. We accounted for demographic differences between groups with regression analysis, but other unaccounted variables likely remain.

Second, despite personal chart review and data collection, we were unable to collect information about certain important variables. In addition to data regarding compliance or medications as discussed previously, we were unable to collect data on postpartum glucose tolerance tests. We excluded patients with preexisting diabetes, but some of our patients likely still had preexisting diabetes. These conditions typically have worse outcomes than gestational diabetes, and their presence may have affected our study findings.

Third, 10-11% of our study population were unable to be included due to a missing paper medical chart. This is very close to the threshold at which a statistical analysis is likely to be biased [25].

The strength of this study is that it is one of the first studies to examine if glycemic targets should be lower than current recommendations for treatment of GDM. We believe that our ethnic mix and patient characteristics would match most cities located within the United States, and thus, our results are generalizable to them. As mentioned before, we also believe another strength is that this study reflects a natural history experiment in real-world clinical practice. Other strengths include personal chart review, data collection, and consistency of practice as all patients were seen at the same high-risk pregnancy clinic and delivered at the same hospital.

Unselected application of lower glycemic targets did not reduce our maternal or neonatal morbidity in GDM patients by a large amount. A true difference may exist, but the reduction in LGA or macrosomia likely is modest and would require a larger sample size to demonstrate the reduction. The costs associated with a higher number of inductions may mean that pursuing lower glycemic targets is not cost-effective. The more efficient strategy may be to identify the subset of GDM patients with the highest risk for fetal overgrowth and treat those pregnancies with lower glycemic targets. Doing so would help reduce further morbidity that persists in GDM pregnancies despite current optimal treatment.

## Figures and Tables

**Table 1:** Descriptive characteristics by glycemic target group.

Demographic characteristic	High-target group (*n* = 396)	Low-target group (*n* = 208)	*p* value
Maternal age, years (mean ± SD)	29.9 ± 5.7	30.9 ± 5.7	**0.047**
Gravida (mean ± SD)	3.1 ± 1.6	2.9 ± 1.9	0.361
Nullipara (*n*, %)	98 (24.7%)	62 (29.8%)	0.189
BMI first visit (mean ± SD)	31.8 ± 11	33.1 ± 9	0.279
BMI at GDM diagnosis (mean ± SD)	33.5 ± 11	35.3 ± 8	0.114
Race/ethnicity (*n*, %)			**<0.01**
Black (*n*, %)	51 (12.9%)	22 (10.6%)	
White (*n*, %)	78 (19.7%)	77 (37.0%)	
Hispanic (*n*, %)	234 (59.1%)	70 (33.7%)	
Asian (*n*, %)	23 (5.8%)	21 (10.1%)	
Other (*n*, 5)	10 (2.5%)	18 (8.7%)	
Chronic hypertension (*n*, %)	24 (6.1%)	20 (9.6%)	0.135
Prior GDM diagnosis (*n*, %)	91 (23.0%)	30 (14.4%)	**<0.01**
Glucola result (mean ± SD)	167 ± 29.4	174.5 ± 33.2	**0.037**
GTT, fasting (mean ± SD)	97.6 ± 19.3	93.7 ± 14.5	**0.038**
GTT, 1 hour (mean ± SD)	198.4 ± 34.0	194.2 ± 29.9	0.25
GTT, 2 hours (mean ± SD)	175.1 ± 32.0	171.9 ± 29.7	0.369
GTT, 3 hours (mean ± SD)	133.9 ± 33.6	129.0 ± 36.8	0.227

**Table 2:** Pregnancy outcomes, by glycemic target groups.

Measured outcome	High-target group (*n* = 396)	Low-target group (*n* = 208)	*p* value
Gestational age at delivery (mean ± SD)	38.6 ± 1.7	38.4 ± 1.5	0.413
<37 weeks (*n*, %)	37 (9.3%)	18 (8.7%)	0.78
≥37 weeks (*n*, %)	359 (90.7%)	190 (91.3%)	0.78
Cesarean delivery (*n*, %)	153 (38.6%)	77 (37.0%)	0.698
Cesarean, intrapartum (*n*, %)	61 (15.4%)	35 (16.8%)	0.653
Induction of labor, secondary to GDM (*n*, %)	81 (20.5%)	82 (39.4%)	**<0.01**
5-minute APGAR > 7 (*n*, %)	385 (97.2%)	204 (98.1%)	0.513
Birthweight (mean ± SD)	3521.7 ± 1848.1	3264.9 ± 558.3	0.051
SGA (*n*, %)	7 (1.8%)	2 (1.0%)	0.435
AGA (*n*, %)	263 (66.4%)	156 (75.0%)	**0.029**
LGA (*n*, %)	126 (31.8%)	50 (24.0%)	**0.046**
Gestational hypertension (*n*, %)	7 (1.9%)	5 (2.4%)	0.595
Preeclampsia (*n*, %)	27 (6.8%)	13 (6.3%)	0.79
Severe preeclampsia (*n*, %)	6 (1.5%)	2 (1.0%)	0.572
Shoulder dystocia (*n*, %)	17 (4.3%)	9 (4.3%)	0.984
Intrauterine growth restriction (*n*, %)	8 (2.0%)	2 (1.0%)	0.333
NICU admission (*n*, %)	51 (12.9%)	33 (15.9%)	0.328
Neonatal hypoglycemia (*n*, %)	46 (11.6%)	43 (20.7%)	**<0.01**
Neonatal hyperbilirubinemia, requiring phototherapy (*n*, %)	20 (5.1%)	14 (6.7%)	0.395
Neonatal RDS (*n*, %)	20 (5.1%)	15 (7.2%)	0.306
Macrosomia (*n*, %)	58 (14.6%)	20 (9.6%)	0.064

**Table 3:** Logistic regression: adjusted odds ratios for low glycemic targets.

Outcomes	Adjusted odds ratio (95% CI)	*p* value
LGA	0.64 (0.34, 1.19)	0.158
Macrosomia	0.63 (0.25, 1.57)	0.318
Neonatal hypoglycemia	1.04 (0.48, 2.25)	0.922
Inductions of labor	**2.54 (1.44, 4.49)**	**0.001**
Intrapartum cesarean deliveries	1.03 (0.52, 2.05)	0.937
Overall cesarean deliveries	0.92 (0.55, 1.53)	0.739

Model: constant+low targets+nullipara+BMI intake+prior GDM+Hispanic.

## Data Availability

The retrospective deidentified clinical data used to support the findings of this study are available from the corresponding author upon request.
